# Editorial: Conference research topic: 16th international symposium on schistosomiasis NEW ('th') presented in superscript

**DOI:** 10.3389/fimmu.2024.1462318

**Published:** 2024-08-16

**Authors:** Marina Moraes Mourão, Roberta Lima Caldeira, Thiago Almeida Pereira, Cristina Toscano Fonseca

**Affiliations:** ^1^ Grupo de Helmintologia e Malacologia Médica, Instituto René Rachou, Fundação Oswaldo Cruz, Belo Horizonte, Brazil; ^2^ Institute for Stem Cell Biology and Regenerative Medicine, Stanford University School of Medicine, Stanford, CA, United States; ^3^ Grupo de Biologia e Imunologia de Doenças Infecciosas e Parasitárias, Instituto René Rachou, Fundação Oswaldo Cruz, Belo Horizonte, Brazil

**Keywords:** International Symposium on Schistosomiasis, host-parasite interactions, vaccines, immunobiology, *Biomphalaria* sp., *Schistosoma* sp, diagnosis, public health

The International Symposium on Schistosomiasis is the foremost scientific gathering dedicated to this significant and neglected disease worldwide. Its 16^th^ edition occurred in Minas Gerais, 2022, a Brazilian endemic state for the disease. The theme of this edition was “Schistosomiasis and Citizenship,” with topics that addressed initiatives for disease control and elimination, the development of tools such as vaccines, medications, diagnostic tests, strategies for controlling the intermediate host, health education, and sanitation actions. In addition, epidemiological, clinical, and parasitological aspects, including the intermediate hosts, were broadly discussed.

The 16^th^ symposium saw a turnout of 329 registered participants from 11 countries and 20 Brazilian states. Five keynote speeches, 98 studies presented as posters, ten panel discussions with 37 talks delivered by invited experts, and 24 selected from abstract submissions. The proceedings of the symposium encompassed 135 abstracts.

This Research Topic aims to widely disseminate some of the studies presented during the event and provide an opportunity for researchers who could not attend to share their study results on the event’s theme. This Symposium edition includes 14 manuscripts, two reviews, and 12 original research articles from 108 authors worldwide.

## Initiative for the control and elimination of schistosomiasis as a public health problem

The WHO’s Guideline on the Control and Elimination of Human Schistosomiasis outlines six recommendations to assist national programs in endemic countries. These recommendations focus on achieving morbidity control, eliminating schistosomiasis as a public health issue, and progressing towards interrupting transmission in line with the 2030 Agenda (1). Subsequently, members of the Oswaldo Cruz Foundation’s Schistosomiasis Translational Program (Fio-schisto) and other experts from Brazil discussed the feasibility of this guideline for the Brazilian settings, published in the article of Menezes et al. The Fio-schisto proposes, for Brazil, interventions in basic sanitation as a priority measure envisioning eliminating schistosomiasis transmission, associated with the Information, Education, and Communication (IEC) strategy and other control measures, actively involving the school community. A two-stage immunological and molecular testing approach was recommended to verify transmission interruption during and after the intervention.

The paper by Wang et al. highlights the importance of education for transmission control. This study aimed to explore the Protection Motivation Theory (PMT) in predicting the likelihood of engaging in protective behavior against *Schistosoma* infection. They showed that in China, behavior intention is a complicated and indispensable part of changes in the population conduct, influenced by professional knowledge, socio-economic status, and personal characteristics. Effective dissemination of knowledge about schistosomiasis should be strengthened to ensure the effectiveness of protective measures against infection and severe disease.

To improve the species-specific recognition of the genus *Biomphalaria*, Araujo et al. used: 1) DNA barcoding methods, Barcode of Life Data System (BOLD) identification criteria and Best Close Match, 2) barcode gap, using the Kimura two-parameter model to calculate intraspecific and interspecific distance, and 3) sequences clustered/grouped into operational taxonomic units: Generalized Mixed Yule-Coalescent, Poisson Tree Processes, Automatic Barcode Gap Discovery, and Assemble Species by Automatic Partitioning. This work generated partial sequences of the *coi* gene and allowed the correct delimitation of most Brazilian *Biomphalaria* species using DNA barcoding and clustering/phylogenetic algorithms. This is a valuable work since there are 11 species of *Biomphalaria* sp. in Brazil, but only three species are of epidemiological importance for schistosomiasis.

## Diagnosis development and evaluation

A new diagnostic test for schistosomiasis is essential for achieving schistosomiasis elimination as a public health problem by 2030 and certifying transmission interruption (1). Scientists worldwide are making significant efforts to develop and evaluate new diagnosis tools. During the symposium, several studies in the diagnosis area were presented. Two of these studies were reported in more detail in this Research Topic, and three other studies using point-of-care diagnosis were published in this Research Topic.

In their manuscript, Mesquita et al. evaluated the accuracy of eleven diagnostic tests for *S. mansoni* infection in a prospective blind study conducted in a low-endemic setting in Brazil. They evaluated point-of-care circulating cathodic antigen (POC-CCA) in the urine, PCR, qPCR, PCR-ELISA tests, and a loop-mediated isothermal amplification (LAMP) test in feces and urine samples. Additionally, they assessed three different ELISA tests without a good index test, thus using a latent class model to determine infection status. This study demonstrates the performance of all tests using different biological samples and proposes three different diagnostic strategies based on two tests: a sensitive ELISA-based test to initially screen for infection, and a confirmatory test based on molecular methods as a second step. This strategy proved more accurate and less expensive than any molecular test alone.

Given the broad distribution of *S. mansoni* infection in endemic countries, qPCR as a diagnostic test relies on infrastructure and logistics. Therefore, isothermal amplification techniques such as the Recombinase Polymerase Amplification (RPA) arise as an alternative. Mesquita et al. developed two RPA assays based on mitochondrial minisatellite DNA detection. The assays differed in their strategies. In the Real-Time RPA, amplification is detected based on the fluorescence emitted during amplification, while it is captured on the test line in the lateral flow format. Both formats performed well using full or half recommended volumes of reagents, were species-specific, and detected one copy of the target gene. No cross-amplification was observed.

To improve diagnosis, surveillance, and mapping of schistosomiasis cases in resource-limiting settings, Zacharia et al. provided a basis for the use of the dried urine spot method and POC-CCA. Collection, storage, and transportation of urine samples can be challenging in resource-limited settings. The authors demonstrated that the filter paper-based dried urine spot method could detect CCA antigens using the POC-CCA test without compromising the integrity of the results and is, therefore, an alternative to testing of fresh or stored samples.

Seeking a diagnostic assay to screen *S. japonicum* infection, Mu et al. developed a gold-immunochromatography assay (GICA) that detected antibodies against the *S. japonicum* saposin antigen (SjSAP-4). This antigen has a low predicted potential for cross-reactivity with antibodies against other parasite flukes and doesn’t cross-react with antibodies against alveolar echinococcosis and trichinellosis. An interesting strategy was applied in the design and laboratory evaluation of the test suggesting its promise as a tool for screening cases in endemic settings and detecting the disease in non-endemic areas.

In the subsequent validation of the GICA test, Mu et al. assessed its performance in 412 individuals from *S. japonicum* endemic areas when compared with Kato-Katz (KK), POC-CCA, two in-house ELISA tests, and the droplet digital PCR assay (ddPCR) performed using feces, urine, serum, and saliva samples. Different sensibility and specificity values were observed depending on the index test used, highlighting the impact of the choice of index texts in diagnosis evaluation.

In diseased diagnosis, Zhong and Jin highlighted the importance of unisexual-schistosome exposure as a neglected phenomenon that should be considered when choosing a diagnostic strategy since unisexual-infected individuals lack the obvious clinical symptoms of the disease and might be less sensitive to praziquantel. In their mini-review, the authors summarized recent advances in our understanding of unisexual schistosomes and host-parasite interactions.

## Host-parasite interaction and functional characterization

Defining host-parasite interactions is crucial to identifying novel strategies to help eliminate schistosomiasis. However, the lack of appropriate tools to study such interactions can be challenging, especially in the intermediate snail host. Duval et al. provided a new tool to study *Schistosoma-Biomphalaria* interactions using fluorescent non-transgenic cell trackers as a non-invasive tool for monitoring different stages of *S. mansoni* within the host without compromising viability and virulence. Combining this tool with vibratome histological techniques allows the visualization of the entire snail without damaging tissue structure. This novel protocol will help to elucidate the nuances of host-parasite interactions in the intermediate host.

The search for novel drugs against schistosomiasis is essential since the treatment relies on a single drug, praziquantel. Coelho et al. explored the functions and expression profile of Protein Arginine Methyltransferases (PRMTs), which catalyze posttranslational modifications, affecting both histone and non-histone proteins. The authors analyzed single-cell RNA-seq data and revealed that most *S. mansoni* (Sm) PRMTs are evenly distributed across various cell clusters in schistosomes. Functional interrogation using knockdown approaches decreased oviposition *in vitro* and *in vivo*. *Ex vivo* analysis revealed structural abnormalities in these worms. This study provides insights into SmCARM1 in *S. mansoni* biology, suggesting its potential as a drug target.

Schistosomes rely on the purine salvage pathway to secure their puric bases, resulting in an energy economy, which could be a choke point for parasite survival. Batista et al. interrogated the function of a gene family regulated by the Smp38 MAP kinase pathway, Hypoxanthine guanine phosphoribosyl transferases 1 and 3 (SmHGPRTases). It was shown that those proteins could be parasite-specific druggable targets, and that all members of the family participate in adenosine uptake. Ex vivo knocked-down in females exhibited immature eggs and impaired ovary development. Therefore, this study supports SmHGPRTases’ importance as target candidates for schistosomiasis and parasite biology.

Continuing the theme of the search for new targets for drugs or vaccine development, Patrick Skelly and Da’dara presented a characterization of the gene encoding the schistosome tegumental acetylcholinesterase (AChE) in three major schistosome species. They demonstrated that schistosomes cleave exogenous acetylthiocholine and detangled the previous annotation of AChE enzymes. *S. mansoni* tegumental AChE (SmTAchE) is different from its human counterpart at a moderate level, and exposure to antibodies targeting SmTAChE impaired schistosomula viability. Thus, they propose it as a vaccine or therapeutic target. Therefore, this work aids in clarifying some of the questions regarding AChEs in this pathogen and highlights new therapeutic targets.

Vaccine development for schistosomiasis remains challenging despite intensive research. Understanding the natural mechanisms of self-cure in animal models such as the rhesus macaque or mice after multiple exposures to a radiation-attenuated cercarial vaccine may provide novel targets for vaccine development. Vance et al. compared the reactivity of sera pools from rhesus macaques after self-curing to four peptide arrays of secreted/exposed proteins from the alimentary tract and tegument of *S. mansoni*. The titer was the primary determinant for the rate of self-cure. Their screening provides the community with a list of candidate epitopes that could be combined to develop an effective vaccine.

## Closing remarks

The 16th International Symposium on Schistosomiasis, held in Brazil, focused on disease control and elimination. It brought together participants from 11 countries to discuss various aspects, including the development of disease control tools and health education. The Research Topic dedicated to the symposium aimed at disseminating selected studies presented during the event to advance disease knowledge and control. Implementing WHO guidelines and improving species-specific recognition of the *Biomphalaria* genus was highlighted. Diagnostic tests for schistosomiasis were evaluated, including the accuracy of different tests and the development of new assays. Host-parasite interactions were studied, leading to the development of a new tool for tracking *S. mansoni* in the intermediate snail host. The functions of PRMTs and SmHGPRTases were explored as potential drug targets, and the gene encoding schistosome tegumental acetylcholinesterase was characterized as a potential vaccine or therapeutic target. Screening of self-cured macaques provided candidate epitopes for vaccine development. The 16^th^ International Symposium on Schistosomiasis and the Pre-Symposium Research Topics (2) hold 48 manuscripts published in different sections of Frontiers, enriching this area of knowledge covering all aspects of schistosomiasis. These studies contribute to the search for new strategies to combat schistosomiasis.

## Author contributions

MM: Writing – review & editing, Writing – original draft. RC: Writing – review & editing, Writing – original draft. TA: Writing – review & editing, Writing – original draft. CF: Writing – review & editing, Writing – original draft.
